# Snare traction-assisted endoscopic submucosal dissection for removal of rare giant laryngeal liposarcoma

**DOI:** 10.1055/a-2307-6213

**Published:** 2024-05-07

**Authors:** Rui Zhao, Jiaxin Yan, Ye-Han Zhou, Yu Bao, Yan Zhang

**Affiliations:** 1 34753 Department of Gastroenterology and Hepatology, West China Hospital of Sichuan University, Chengdu, China; 2 92293 Department of Endoscopy, Sichuan Cancer Hospital and Institute, Chengdu, China; 3 92293 Department of Pathology, Sichuan Cancer Hospital and Institute, Chengdu, China

A 78-year-old man presented with a foreign body sensation in the throat for 1 year. Endoscopy revealed a large pedunculated tumor in the throat, extending deep into the mid-thoracic esophagus ( Fig. 1 ). Computed tomography (CT) of the throat showed a column-like region with a density similar to fat located within the larynx and esophageal entrance to the mid-thoracic lumen, without significant contrast enhancement ( Fig. 2 ).

**Fig. 1:**
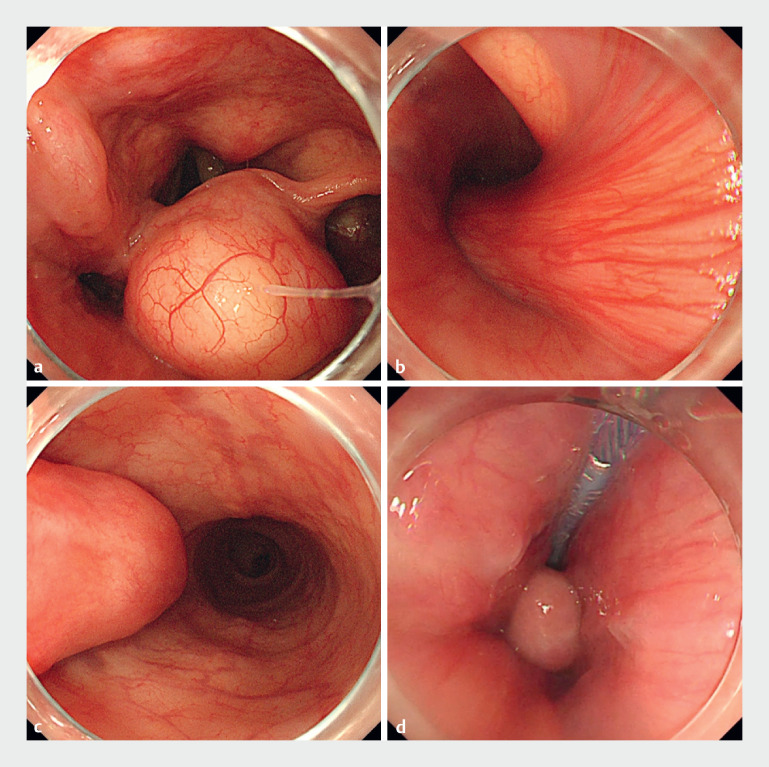
Endoscopic findings. **a** Tumor in the larynx. **b** Esophageal entrance. **c** Mid-esophagus. **d** Snare traction.

**Fig. 2:**
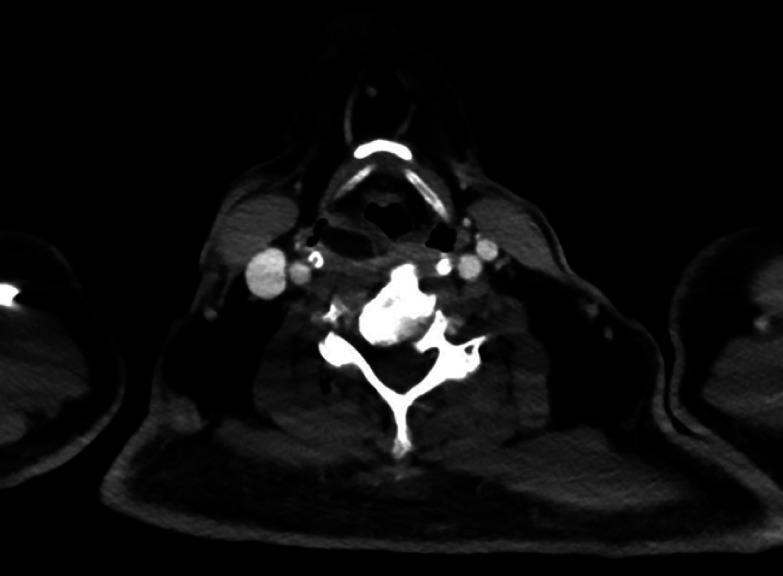
Computed tomography showing column-like region with a fat-like density in the larynx, without significant contrast enhancement.

We performed endoscopic submucosal dissection (ESD) using a snare to expose and retract the base of the lesion ( Video 1 ). The entire operation lasted 53 min. The tumor was removed through the mouth using the snare ( Fig. 3 ). No adverse events or complications were observed.

Endoscopic submucosal dissection (ESD) was performed to remove a giant laryngeal liposarcoma.Video 1

**Fig. 3:**
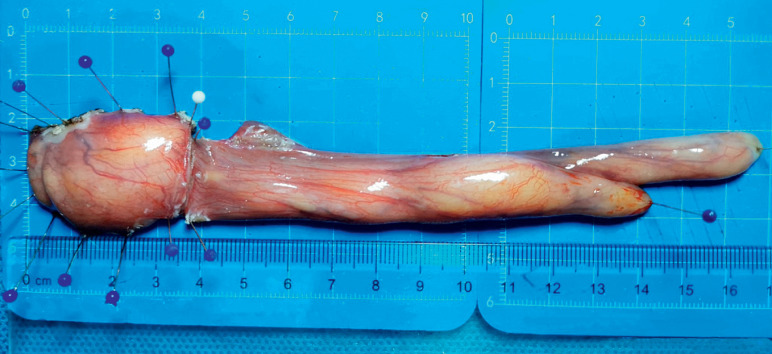
The specimen measured 16.5 cm in length and 2.7 cm in width at its widest point.

Histological analysis revealed a well-differentiated liposarcoma. Furthermore, fluorescence in situ hybridization demonstrated significant *MDM2* gene amplification ( Fig. 4 ).

**Fig. 4:**
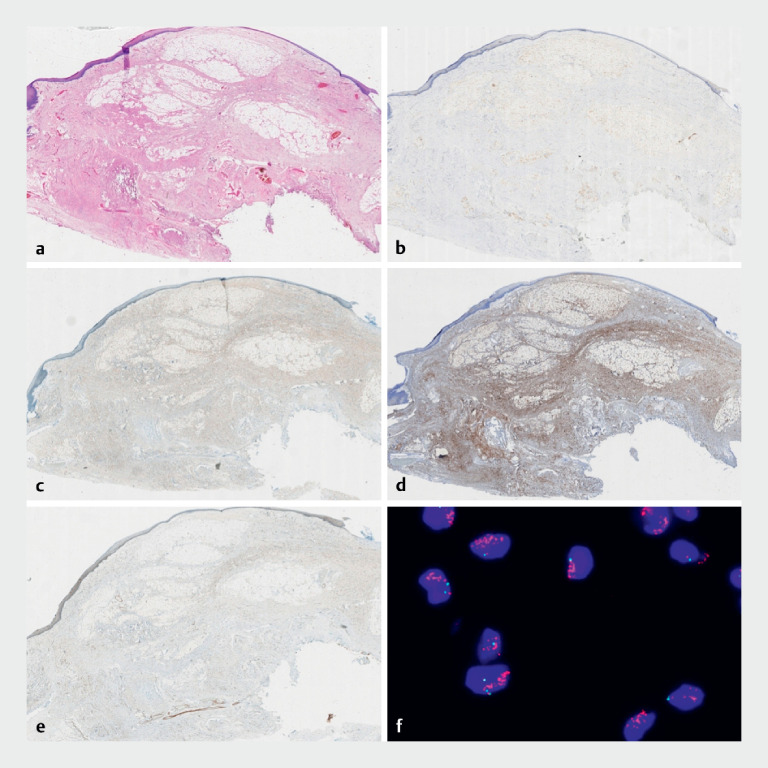
Histological analysis revealed a well-differentiated liposarcoma. **a** Hematoxylin and eosin-stained images (× 40). **b–e** The tumor cells were S100(+) ( **b** ), CDK4(+) ( **c** ), P16(+) ( **d** ), and MDM2(+) ( **e** ). **f** Fluorescence in situ hybridization showed that 67% of cells had an amplification of MDM2 copy numbers.

Well-differentiated liposarcomas are frequently encountered in the esophagus and hypopharynx 1 , with rare occurrences in the larynx. While previous reports have described the removal of liposarcomas from the hypopharynx using ESD or surgical resection 2 3 , the excision of laryngeal liposarcomas using ESD has not been reported. Here we present the first reported case of complete excision of a laryngeal liposarcoma using ESD.

The management of large neoplasms can be improved with the use of a snare for applying effective traction. However, its application for the treatment of giant tumors in the larynx has not been documented. In the confined pharyngeal and laryngeal spaces, intraoperative snare-assisted traction of the lesion aids in generating adequate space and offering a clear view, thus enabling complete tumor excision at the basal cut margin. This case demonstrates the efficacy of snare traction-assisted ESD as a minimally invasive, safe, and feasible treatment method for large pedunculated laryngeal tumors. Regular postoperative follow-up is necessary to monitor for local recurrence.

Endoscopy_UCTN_Code_TTT_1AO_2AG_3AD

## Notes

Endoscopy E-Videos https://eref.thieme.de/e-videos
*E-Videos* is an open access online section of the journal *Endoscopy* , reporting on interesting cases and new techniques in gastroenterological endoscopy. All papers include a high-quality video and are published with a Creative Commons CC-BY license. Endoscopy E-Videos qualify for HINARI discounts and waivers and eligibility is automatically checked during the submission process. We grant 100% waivers to articles whose corresponding authors are based in Group A countries and 50% waivers to those who are based in Group B countries as classified by Research4Life (see: https://www.research4life.org/access/eligibility/ ). This section has its own submission website at https://mc.manuscriptcentral.com/e-videos .
